# Development of Hydrogels and Biomimetic Regulators as Tissue Engineering Scaffolds

**DOI:** 10.3390/membranes2010070

**Published:** 2012-02-14

**Authors:** Junbin Shi, Malcolm M. Q. Xing, Wen Zhong

**Affiliations:** 1 Department of Textile Sciences, Faculty of Human Ecology, University of Manitoba, Winnipeg, MB R3T 2N2, Canada; Email: shijunbin88@hotmail.com; 2 Department of Mechanical Engineering, University of Manitoba, Winnipeg, MB R3T 2N2, Canada; 3 Manitoba Institute of Child Health, Winnipeg, MB R3E 3P5, Canada; 4 Department of Biochemistry and Medical Genetics, University of Manitoba, Winnipeg, MB R3T 2N2, Canada; 5 Department of Medical Microbiology, Faculty of Medicine, University of Manitoba, Winnipeg, MB R3T 2N2, Canada

**Keywords:** hydrogel, bone, tissue engineering

## Abstract

This paper reviews major research and development issues relating to hydrogels as scaffolds for tissue engineering, the article starts with a brief introduction of tissue engineering and hydrogels as extracellular matrix mimics, followed by a description of the various types of hydrogels and preparation methods, before a discussion of the physical and chemical properties that are important to their application. There follows a short comment on the trends of future research and development. Throughout the discussion there is an emphasis on the genetic understanding of bone tissue engineering application.

## 1. Introduction

Defining tissue engineering (TE) as “an interdisciplinary field that applies the principles of engineering and life sciences toward the development of biological substitutes that restore, maintain, or improve tissue function”, Langer and Vacanti identified three general strategies for tissue engineering, that is the use of (1) isolated cells or cell substitutes, (2) tissue-inducing substances, and (3) cells placed on or within matrices [1]. They constitute the prevalent method of using biodegradable polymer matrices as a carrier for cell transplantation [2], whereby cell populations isolated from tissue are seeded on polymer scaffolds to generate cell/matrix constructs for *in vivo* implantation [3]. Thus, biomaterials function as polymer scaffolding to help cell organization and growth and allow nutrients to be transported to the transplanted cells. That is, the TE strategies emphasize the basics of cell growth and differentiation, *in vitro* control of tissue development, *in vivo* synthesis of tissues, the use of biomaterials as scaffolds in tissue engineering, transplantation issues and applications in the cardiovascular system, the gastrointestinal system, the kidney, reconstruction of cornea and pancreas, growth of cartilage and bones, nervous tissue regeneration, and dental and skin applications [4]. 

In native tissue, the extracellular matrix (ECM) provides structural integrity and binding support to the cells, and the cells are constantly remodeling the ECM [5]. The extracellular matrix includes the interstitial matrix and the basement membrane. The former is present between various animal cells and filled with gels of polysaccharides and fibrous proteins that act as a compression buffer against the stress placed on the ECM [6]. Scaffolds used in tissue engineering mimic the natural extracellular matrix (ECM) and provide support for cell adhesion, migration, and proliferation [7]. 

Hydrogels are selected as scaffolds and employed as multidimensional [two dimensional (hydrogel membrane) or three dimensional (hydrogel block)] cell culture platforms with the necessity that they respond to or control the cellular environment [8]. Efforts should be made to ensure that materials for hydrogels are free from such problems as mechanical materials failure, materials-associated infection, and immunogenic reaction to implanted materials [9]. There may be other requirements for specific applications. For example, a hydrogel should, when transplanted, be able to promote cell adhesion and tissue regeneration and to assist the exchange of metabolites (oxygen and nutrition) for a 3D cell culture.

For the application in implantation, gels have been designed to be biodegradable so that they can be degraded within and ultimately absorbed by the body, all at the same time as tissue is “regenerated”. Hydrogels are typically biocompatible, neither provoking immune response nor causing inflammation. Hydrogels have thus been found to possess tissue-like properties as they can be successfully used to encapsulate cells and mimic a cell scaffold environment [9]. 

## 2. Preparation and Characterization of Hydrogels

Hydrogels are polymeric materials that swell in water and retain a significant fraction of water within the three dimensional network (cross-linked structures) without dissolving [10,11]. This section is about the various preparation and characterization processes for bio-hydrogels, *i.e.*, (1) what they are or are made from, what their chemical and physical properties are, and how these are related to the functions they are expected to perform, and (2) how we can come to know all these characteristics.

### 2.1. Materials for Hydrogels

Biomaterials for hydrogels are used or designed to elicit specific cellular functions and to direct cell-cell interactions both in implants that are initially cell-free and may serve as matrices to contribute to tissue regeneration, and in implants to support cell transplantation [12]. Materials for hydrogels can be classified according to their source as natural or synthetic. Their biocompatibility and biodegradability are essential to their application in tissue engineering, and will therefore be given greater attention. 

Naturally occurring biomaterials (collagen, chitosan, hyaluronic acid, fibrin, gelatine, *etc*.) are expected to most closely simulate the native cellular environment. Uses of these are limited, however, by such factors as large batch-to-batch variation upon isolation from biological tissues, strict requirements for specific biomechanical properties, and so on, not to mention that some of them are rather costly [3,9]. 

#### 2.1.1. Hyaluronic Acid (HA)

Hyaluronic acid is part of the ECM [13], and a naturally occurring linear polysaccharide that is abundant in the vitreous and synovial fluid and plays important roles in wound healing [14], cell differentiation and cell motility [15]. Research on stem cell culture indicates that function of the HA is essential also to the control of self-renewal of human embryonic stem cells [16]. It is highly biocompatible, suitable for modification with drugs and other effector molecules [17]. HA and its derivatives have been used to compose drug delivery systems consisting of a wide variety of drugs and cell encapsulation [11,17].

HA is composed of beta (1–4) linked 2-acetamide-2-deoxy-*D*-glucose and beta (1–3) linked *D*-glucuronic acid, and is known to be non-antigenic, noninflammatory and generally non-tissue reactive [18]. HA can be degraded by HAse *in vivo*, which is ubiquitous in cells and in serum [19]. For the purpose of *in vitro* HA hydrogel study, different methods have been developed for measuring the hydrogel degradation, by such means as monitoring the release of uronic acid (a degradation component of HA) from a matrix, or monitoring the loss of weight [20,21,22].

The crosslinkage of HA hydrogel can be formed as a result of polyvalent hydrazide cross-linking, disulfide cross-linking, photo-crosslinking, or enzymatical crosslinking [11,17,22,23]. Pure HA hydrogel is a nonadhesive hydrogel for cells, and the promotion of cell adhesion can be achieved by mixing pure HA hydrogel with gelatin microparticle [24,25].

#### 2.1.2. Chitosan

Another type of polysaccharide, chitosan has been widely used for biomedical applications, such as cell culture platforms [26,27], drug carriers [28] and non-viral gene delivery [29]. Chitosan is derived from chitin with a linear structure, consisting of *β*-(1–4)-linked 2-amino-2-deoxy-*D*-glucose and 2-acetamido-2-deoxy-*D*-glucose units [30]. Chitosan can be easily modified, usually via primary amine groups [31]. However, Chitosan can only be dissolved in an acetic environment, which limits its applications in injectable cell culture scaffolds. A common solution is to partially *N*-succinylate chitosan to make it dissolvable in a neutral solution [32]. Chitosan has excellent biodegradability as well [28]. Lysozyme is used to degrade chitosan for *in vitro* degradation evaluations [33]. Chitosan polymer chains can be crosslinked by glutaraldehyde or genipin to form chitosan hydrogel [34,35]. Other methods have also been developed to from chitosan hydrogels, such as maleic chitosan [36].

#### 2.1.3. Alginate

Alginate is block polymer composed of (1–4)-linked b-*D*-mannuronic acid (M units) and a-*L*-guluronic acid (G units) monomers, the ratio varying between M and G units [37]. Alginate polysaccharides covalently modified with RGD-containing cell adhesion legends are widely used for the settlement and attachment of cells. In addition, calcium alginate hydrogel surfaces coupled with GRGDY peptides can be fabricated to achieve cellular interaction [38]. They are well known for their uses in mineralized polymeric matrices, which justifies their application in bone tissue regeneration [39]. However, it is difficult to have alginate degraded *in vivo* and in a short time [40]. 

#### 2.1.4. Collagen (and Gelatin)

Collagen is naturally occurring proteins in the form of elongated fibrils and found in mammals [41,42]. It is the main component of connective tissues such as tendons, ligaments and skin, and is up to 35% of the body protein content, *i.e.*, the most abundant protein in mammals [43]. Gelatin is collagen that has been hydrolyzed under basic and high temperature conditions [44]. Collagen or Gelatin can be degraded by enzyme collagenase *in vivo* [45]. The methods used to form the collagen or gelatin hydrogel are quite similar to those for HA, such as disulfide cross-linking, photo-crosslinking, and enzymatical crosslinking [46,47,48].

Synthetic biomaterials are favored as scaffold materials because their physical and biologic properties can be modified and they can be reproduced in similar and large quantities. The major classes of synthetic biomaterials are the glycolic acid derivatives, lactic acid derivatives, and other polyester derivatives [9]. 

#### 2.1.5. Polyethylene Glycol (PEG)

Widely used in human medicine, PEG resists protein adsorption and is therefore endowed with unique nonfouling properties because of its nonadhesivity towards proteins and cells [8,49]. 

PEG is non-ionic and soluble in water. PEG diacrylate is produced as a result of the modification of PEG with acrylate to get carbon double bone which will serve as a gelation functional group [36]. Application of PEG or its diacrylate derivative (PEGDA) to tissue engineering [49,50,51,52] is limited by their inability to support cell spreading due to their being non-adhesive to protein and the absence of cell adhesion ligand [49,53].

Pure PEG (molecular weight <20,000) is chemically stable under physiological conditions [40]. However, after modification some polymers based on PEG can be biodegradable, as instanced by oligo(poly(ethylene glycol) fumarate) (molecular weight ~10,000) [54].

The many crosslinking methods for PEG hydrogels, such as the thermal radical initiation system [54] and disulfide crosslinking system [23], have been extensively investigated so that nowadays both chemical hydrogels and physical hydrogels are available [55].

#### 2.1.6. Polyvinyl Alcohol (PVA)

Similar to PEG, PVA is another type of hydrophilic synthetic material. Its excellent biocompatibility makes it one of the most popular materials in medical applications [56]. However, similar to PEG, PVA does not support cell spreading and adhesion. Modification therefore is required before its use in tissue engineering [57]. PVA hydrogel is not biodegradable, but its acrylate modified form is degradable [58]. Other methods, like incorporation of biodegradable crosslinkers into PVA hydrogel, is also developed [59].

#### 2.1.7. Poly(2-Hydroxyethyl Methacrylate)

P(HEMA), the homo-polymer or co-polymer from 2-hydroxyethyl methacrylate (HEMA), has been used as implant materials for a long time [60]. The contact lens is of the classic chemical cross-linking hydrogels developed by Wichterle and Lim as a result of the copolymerization of hydroxyethyl methacrylate (HEMA) [61].

The p(HEMA) hydrogel is non-biodegradable, a property that restricts its application in tissue engineering, but is advantageous for its micro porous structure (a preferable morphology for tissue engineering scaffold) as a result of the polymerization-induced phase separation polymerization [62].

#### 2.1.8. Poly(Amido-Amine)

Poly(amido-amine)s (PAAs) are a class of polymers characterized by the presence of amido and tertiary amino groups regularly arranged along the macromolecular chain. The backbone of poly (amido-amine)s are usually synthesized by poly addition of primary monoamines, or bis(secondary amines), to bis-acrylamides [63].

PAAs can be easily modified during synthesis by the introduction of functional co monomers [64]. For example, a new PAAs was synthesized to contain the disulfide linkage in the main chain by means of stepwise polyaddition of 2-methylpiperazine to *N*,*N*-bis(acryloyl)cystamine (BACy1) or *N*,*N*-bis(acryloyl)-(*L*)-cystine (BACy2). This kind of functionalized PAAs exhibit their good biodegradability when they are degraded by reduction of the disulfide group under physiological conditions. Another example is the biomimetic poly(amidoamine) hydrogels for cell culture that evolve from the incorporation of 4-aminobutylguanidine (agmatine) moieties to create RGD-mimicking repeating units for promoting cell adhesion [64].

PAAs are synthetic polymers endowed with biologically interesting properties, such as being highly biocompatible, free from toxicity, and biodegradable. When it is positively charged, however, because of the positive charge of tertiary amine group and the absent of negative charge group, this kind of PAAs will show cytotoxicity, which is partially dependent on its positive charge arrangement and ions density [65,66,67]. Most PAAs for biomedical application have carboxyl groups along the main chain to decrease cytotoxicity and form amphoteric PAAs [68].

#### 2.1.9. PEO-PPO-PEO Triblock Copolymer

PEO-PPO-PEO is a triblock copolymer consisting of poly (ethylene oxide) (PEO) and poly (propylene oxide) (PPO). It attracts interests in biomedical applications as it forms a thermo-reversible gel that shows gelation at around the body temperature (37 °C) via PPO segments aggregation [69,70] Furthermore, the block composition (PEO/PPO ratio) and the molecular weight can be used to control the final properties of the products so as to meet the specific application needs [71]. However, it has the same issues in biodegradation [72] and cell attachment [73] when it is applied in tissue engineering. The solutions to these issues are similar to those related to PEG or PVA.

Combination of natural and synthetic hydrogels has been utilized to overcome shortcomings of pure natural and synthetic materials. Biomaterials combined in different ways have been developed to generate hydrogels with biological properties (e.g., hydrophilicity, cell-adhesiveness, degradability), biophysical properties (e.g., porosity, branched vasculature), and mechanical ones (e.g., stiffness, viscoelasticity) [5]. 

#### 2.1.10. Cross Linkable Group Modification

Modification of natural polymers for creating cross linkable functional groups is a basic step for hydrogel design and further application. Arylate is one of such functional groups for such modification. HA-acrylate can form a stable covalent crosslink hydrogel via a free-radical mechanism between carbon-carbon double bones (a covalent bond where two pairs of electrons are shared between the atoms rather than one pair) converting the double to a single bond and forming single bonds to join the other monomers. One of such efforts is to modify HA with methacrylic anhydride by adding ethyl eosin and triethanolamine as initiator, to result in the synthesis of a photocrosslinked polysaccharide hydrogel by irradiating using an argon ion laser [11]. 

Enzyme induced crosslink is also widely used to form hydrogels because of its low cytotoxicity. This crosslink system requires modification of natural polymer with an enzyme catalytic group. In one instance hydroxyphenylpropionic (HPA) acid was treated with NHS/EDC to link it to gelatin by reacting with tyrosine residues of the gelatin. Those conjugates can be catalyzed by hydrogen peroxide (H_2_O_2_) and horseradish peroxidase (HDP), and then crosslinked with each other to form gels because phenols can be crosslinked through either a more common *C*-*C* linkage between the ortho-carbons of the aromatic ring or a *C*-*O* linkage between the ortho-carbon and the phenolic oxygen. The stiffness of the hydrogels was readily tuned by varying the H_2_O_2_ or HDP concentration [48].

Other modification processes (e.g., disulfide linkage) can be affected by modifying a natural polymer with 3,3’-dithiobis(propionic hydrazide) (DTP) or cystine to form thiolated gelatin or HA macromer [20,74].

#### 2.1.11. Poly(PEG-Co-Peptides) Conjugate

Cell anchorage is a strict requirement for the survival of most cell types, and it orchestrates critical roles in many cellular functions including migration, proliferation, differentiation, and apoptosis. Cell interaction with biomaterials is mediated through transmembrane receptors which recognize adhesion molecules at the materials surface [38].

PEG is a cyto-nonadhesive hydrogel and needs to be modified with cell adhesive peptides if it is to be used as a cell culture platform. Arg-Gly-Asp (RGD), a component of cell binding protein targeting αv-integrins, is widely used to promote cell adhesion [75,76]. RGD peptides was [49] incorporated into PEG diacrylate hydrogel through aminolysis of the *N*-hydroxy succinimide ester of acrylic acid by the a-amine terminus of the peptide sequence to produce an amide linkage between the peptide and the acrylic group.

PEG modified with polylysine [77] which serves as a functional cell attachment group for micro-vessel scaffolds is developed by activating PEG with *N*,*N*′-carbonyldiimidazole and then forming the gel by the reaction between free-amine groups in Poly-*L*-lysine hydrobromide [58].

### 2.2. Formation of Cross-Links

Cross-links in hydrogels are formed by covalent or ionic bonds. Weaker forces such as van der Waals forces and hydrogen bonds can also serve as cross-links, resulting in the formation of swollen networks that will behave as hydrogels. Finally, semicrystalline, uncross-linked hydrophilic polymers may form hydrogels upon swelling since the crystallites act as physical cross-links and do not dissolve in water [10]. They are called “physical” or “reversible” gels when the network is formed by molecular entanglement or by non-covalent force, and called “permanent” or “chemical” gels when a covalently cross-linking network is present [78].

#### 2.2.1. Preparation of Hydrogels by Chemical Cross-Linking

Cross-links can be formed by means of chemical reaction initiated by heat, pressure, change in pH, or radiation [7]. As previously mentioned, the contact lens is a classic chemical cross-linking hydrogel developed by Wichterle and Lim, based on copolymerization of hydroxyethyl methacrylate (HEMA) with the crosslinker ethylene glycol dimethacrylate (EGDMA) [61]. This hydrogel is created by free radical chain polymerization which can be initiated by light, heat, or redox [78].

Reviewed here are the two major methods to initiate free radical chain polymerization for biomaterials: photo-initiated polymerization and radical-initiated polymerization for the preparation of hydrogels. The former is instanced by the modification of hyaluronic acid (HA) with methacrylate functional groups to result in the covalent cross-linking in the presence of a radical-initiated polymerization system. A concrete instance is the modification of HA with methacrylic anhydride to produce a synthetic, photocrosslinked hydrogel [11]. One more instance is the improvement of a polysaccharide hydrogel by using 2-Hydroxy-4′-(2-hydroxyethoxy)-2-methylpropiophenone (Irgacure 2959) as the ultra violet light-sensitive photoinitiator to decrease cytotoxicity, and to encapsulate human embryonic stem cells and control cell proliferation and differentiation [16,36]. Irgacure 2959 was reported to have minimal toxicity towards six cell lines when it was used for cell encapsulation [79]. Upon absorption of UV light, Irgacure 2959, as a free-radical donor, is cleaved into two primary radicals which then react with the vinyl (C=C) groups of modified hyaluronic acid to initiate radical polymerization [80]. 

Radical-initiated polymerization can be achieved by thermal radical initiation reactions. One instance is the design of an injectable glucose sensitive microbead hydrogel by using a host macromer with bi-functional reactive group (vinyl groups), and adding sodium persulfate (SPS) as an initiator and *N*,*N*,*N*′,*N*′-tetramethylethylenediamine (TEMED) as a catalyst [81]. Researchers have now proved the feasibility of this initiation system for its negative cooperative effect to cytotoxicity in these injectable hydrogels [82]. They have also demonstrated the cytocompatiblity of the initiator concentration used in the APS/TEMED initiation system for MSCs in OPF hydrogels [83].

Diverse approaches have been utilized to produce in situ hydrogels with tunable properties using different triggers such as pH, temperature, light, and targeting biomolecules, as instanced by this facile approach to the creation of a “living” controlled *in situ* gelling system based on a thiol-disulfide exchange reaction by changes in the pH of the pre-gel solution [84]. Another example is the self-setting injectable hydrogels that can be triggered by a decreased pH. Silated macromolecules, such as silated hydroxyl propylmethylcellulosc have been used for such purposes [85]. Silated macromolecules can be self-crosslinked by silanol condensation to form hydrogels [86].

#### 2.2.2. Preparation of Hydrogels by Physical Cross-Linking

Physical hydrogels are not homogeneous, because clusters of molecular entanglements, or hydrophobically- or ionically-associated domains, can create inhomogeneities. Free chain ends or chain loops also represent transient network defects in physical gels [78]. 

Electrostatic between a polyelectrolyte and a multivalent ion of the opposite charge accounts for the formation of a physical hydrogel known as “ionotropic” hydrogel [78]. For example, alginates are naturally derived anionic polysaccharides and used as hydrogels by adding calcium ions as the opposite charge [87]. Divalent cations like Ca^2+^ cooperatively bind between the G blocks of adjacent alginate chains, creating ionic interchain bridges which cause gelling of aqueous alginate solutions [38]. 

With the development of nanotechnology, supramolecular self-assembly has attained keen interest in science [88]. Trials have been made adding multivalent ions of the opposite charge to the heated and cooled peptide amphiphile solution to cover and weaken the ionic force, thus breaking the balance between the hydrogen bond, ionic force or van der Waals forces, and to have monomers or macromers aggregated to form the gel [89]. This is instanced by the design of an injectable b-Hairpin peptide hydrogel, which can be triggered by shielding its ionic repulse force in a salt environment, and in which the peptides are capable of killing Methicillin-Resistant Staphylococcus aureus [90]. Besides the ionic trigger, enzymes also can serve as triggers to achieve biocatalytic self-assembly and gelation. A number of related precursors based on aromatic peptide amphiphiles were synthesized to self-assemble supramolecular hydrogel initiated by subtilisin, a hydrolytic enzyme from Bacillus licheniformis, which hydrolyses the methyl ester to form a peptide derivative that will cause a decrease in hydrophilic force. Recent research has shown that the structures can potentially be controlled by appropriate assembling conditions [91]. 

### 2.3. Properties and Characterization of Hydrogels

The materials for and gelation methods of hydrogels affect the physical and chemical properties of the final products. Hydrogel hydrophilic networks have a high affinity for water but are prevented from dissolving due to their chemically or physically crosslinked network. The polymer network absorbs water molecules, resulting in the gradual swelling of the hydrogel [92]. The character of the water in a hydrogel will determine the overall permeation of nutrients and gas into and cellular products out of the gel [21]. 

When a dry hydrogel is immersed in an aqueous environment, the water uptake process consists of quite a few steps. At the beginning, the polar, hydrophilic group gets hydrated by water molecules to lead to the formation of “primary bound water”; hydrophobic groups thus exposed interact with water molecules, leading to hydrophobically-bound water or “secondary bound water”; the swelled network will imbibe additional water due to the osmotic driving force of the network chains towards infinite dilution; this additional swelling is opposed by the covalent or physical crosslinks, leading to an elastic network retraction force; at last, the hydrogel will reach an equilibrium swelling level and begin to disintegrate and dissolve if the network chains or crosslinks are degradable. The additional swelling water is called “free water” or “bulk water”, and is assumed to fill the space between the network chains, and/or the center of larger pores, macro-pores or voids. It is believed that a gel used as a tissue engineering matrix may never be dried, because the total water in the gel is comprised of both “bound” and “free” water [78]. 

Before hydrogel formation, it is critical to monitor the rheological properties of the polymers, including the viscosity of the precursor solution, gelation time, hydrogel pore size, steady shear and dynamic oscillatory information [86].

After gealtion, the most important parameters used to characterize the network structure of hydrogels are the polymer volume fraction in the swollen state, the molecular weight of the polymer chain between two neighboring crosslinking points, and the corresponding mesh size. Parameters relating performance of hydrogels are: swelling behavior, interior morphology, mechanical properties, biodegradation, cytotoxicity, and cell viability, as well as chemical properties, as discussed below.

#### 2.3.1. Swelling Behavior

The polymer volume fraction in the swollen state being a measure of the amount of fluid imbibed and retained by the hydrogel [7], the swelling property to some degree characterizes water thus held in hydrogels, as indicated in a 2010 study. A hydrogel was cast, soaked in ethanol for 48 hours, and then allowed to air dry. When properly dried, the material was placed in a phosphate buffered saline (PBS) solution at 37 °C and allowed to swell for 16 hours. Readings were taken every 30 minutes for the first four hours, then every hour for the next four hours, then every two hours for the remaining eight hours. Gel fraction of each hydrogel sample was measured by determining the weight of the gel before and after soaking in the ethanol. After the initial 16 hours of swelling the samples were monitored for degradation. Weights were taken every 24 hours for the next 30 days. A fresh amount of PBS, previously equilibrated at 37 °C, was employed every 24 hours during the time of measurement to replace the used ones [93].

The mass swelling percentage of the hydrogel was calculated from the following relation [94]:

%*S =* [(*m_t_ − m_0_*)*/m_0_*]× 100 (1)

where *m_0_* is the mass of the dry gel and m_t_ mass of swollen gel at time *t*. 

The swelling rate constant of the hydrogel was calculated from the following relation:

Percentage mass swelling = *k_s_t*^0.5^ (2)

where *k_s_* is the swelling rate.

The initial swelling data were fitted to the exponential heuristic equation.

*F* = *M_t_*/*M_∝_* = *kt^n^* (3)

where *F* is the fractional uptake; *M_t_/M_∝_*, when *M_t_* is the amount of diffusant sorbed at time *t*, *M_∝_* is the maximum amount absorbed; *k* is a constant incorporating characteristics of macromolecular network system and the penetrant; *n* is the diffusional exponent, which is indicative of the transport mechanism. The equation is valid for the first 60% of the normalized solvent uptake. For Fickian kinetics in which the rate of penetrate diffusion is rate limiting, *n* = 0.5, whereas values of n between 0.5 and 1 indicate the contribution of non-Fickian processes such as polymer relaxation. Diffusion coefficients are important penetration parameters of some chemical species to polymeric systems. Using *n* and *k*, the diffusion coefficient (*D*) of the solvent in the matrix can be calculated using the following equations:

*K* = 4[*D*/π*r*^2^]^n^ (4)

4*D^n^* = *k*(πr^2^)^n ^ (5)

*D*^n^ = (*k*/4)( π*r*^2^) (6)

where *D* is the diffusion coefficient and *r* the radius of gel disc.

#### 2.3.2. Gel or Gel/Cell Construct Morphology

The hydrogel is of a cross-linked network. A scanning electron microscope (SEM) is utilized by some researcher for the observation of gel morphology [36,62,77,95,96,97]. The sample for SEM should, first, be frozen quickly to best keep the original morphology upon the samples having reached their maximum swelling ratio in dd-water at room temperature after 24 hours and then freeze dried in a Freeze Drier until all water is sublimed. The freeze-dried hydrogel specimens are then cut and fixed on aluminum stubs and then coated with gold for interior morphology observation with a scanning electron microscope [36]. 

Because dehydration for SEM to some degree leads to artifact in the highly water-saturated gels, their morphology can be better viewed by cryosectioning the gels followed by staining with fluorescein isothiocyanate (FITC), and visulated under fluorescence or confocol microscopy [77].

SEM can also show cell morphology attached to the hydrogel when it is applied to tissue engineering. The method of treating samples can be quite different because cell morphology is subject to changes when going through the freeze drying process. Accordingly, in some studies hydrogels seeded with cells were washed with PBS, fixed with 4% glutaraldehyde or 10% buffered formalin, and dehydrated by the use of graded ethanol followed by the addition of hexamethyldisilizane. Cellular constructs were sputter coated with gold and observed under the SEM [77,98,99].

Backscattered electron microscopy (BSE-SEM) is commonly used for characterizing repaired bone sections by showing the mineralized tissues at the implant-bone interface [100,101]. Furthermore, BSE-SEM provides the mineralized composition and density of bone regenerated from nonautogenous grafts in bone tissue engineering [102,103].

#### 2.3.3. Mechanical Properties

The key structural parameter determining the material modulus and diffusional characteristics is the network crosslinking density. Many means are available for bulk or local gel mechanical measurement, such as the Dynamic Mechanical Analyzer for bulk gel mechanical measurement (DMA), atomic Force Microscopy (AFM) or Tracer Particle Microrheology (TPM) for the measurement of local gel and cell mechanical changes [8].

Researchers have been able to determine the compressive modulus of the various swollen hydrogels on a mechanical tester using a parallel plate apparatus and loading of 10% of the initial thickness per minute (~200 mm/min). Samples for mechanical testing (n = 5 per composition) are cylindrical (~2 mm height, ~7 mm diameter) and are compressed until failure or until 60% of the initial thickness was reached. The modulus is determined as the slope of the stress versus strain curve at low strains(<20%) [21]. 

Mechanical testing can also be performed on a DMA Q800 Dynamic Mechanical Analyzer in a “controlled force” mode, whereby the swollen hydrogel samples in the shape of a circular disc are submerged in distilled water and mounted between the movable compression and fluid cup; a compression force from 0.01 to 0.05 or 0.30 N (depending on the gel strength) at a rate of 0.02 or 0.05 N/min was applied at room temperature; the compression elastic modulus (E) of the swollen hydrogel can be extracted by plotting the compressional force versus strain [104].

In a self-assembly physical hydrogel, AFM is used for to show the gel nanostructure formation and nucleation and early-stage structure growth [91,105]. 

#### 2.3.4. Cytotoxicity and Cell Viability

When cells are seeded to gels in 2D, cytotoxicity is largely related to the chemical structure of the hydrogel; under three dimensional situations, that is, when the cells are encapsulated in the hydrogel, cell viability can also be affected by poor nutrient and gas exchange because of the inappropriate cross-linking density [21]. Assays generally used to measure hydrogel cytotoxicity and cell viability are described as follows.

MTT assay is a popular approach to evaluate cell viability on hydrogels [20,24,36]. MTT (3-(4,5-Dimethylthiazol-2-yl)-2,5-diphenyltetrazolium bromide, a yellow tetrazole) is reduced to purple formazan in living cells [106]. A solubilization solution (usually either dimethyl sulfoxide, an acidified ethanol solution, or a solution of the detergent sodium dodecyl sulfate in diluted hydrochloric acid) is added to dissolve the insoluble purple formazan product into a colored solution. The absorbance of this colored solution can be quantified by measuring at a certain wavelength (usually between 500 and 600 nm, an absorbance maximum at 490–500 nm in phosphate-buffered saline) by a spectrophotometer. The absorption maximum is dependent on the solvent employed [107]. 

Researchers have managed to assess the viability of photoencapsulated fibroblasts immediately after encapsulation and after 1 week of in vitro culture using a commercially available MTT viability assay (ATCC, 30–1010 K). For this assay, 100 µL of the provided MTT reagent (tetrazolium salt solution) is added directly to the wells containing the constructs (n = 3 per macromer solution) and placed in an incubator at 37 °C for 4 hours. The purple formazen produced by active mitochondria is solubilized by construct homogenization in 1 ml of the provided detergent solution and orbital shaking for 2 h. The absorbance of these solutions is then read at 570 nm (SpectraMax 384) (Molecular Devices, Sunnyvale, CA, USA) [21].

Live/Dead assays are cytotoxicity and viability assays too. Live/Dead fluorescent staining (Invitrogen) can be used to test osteoblasts for viability and proliferation at Day 1, 3 and 7. The live (green) and dead (red) cells are observed after 30 min incubation and sufficient PBS wash. For quantitative evaluation, WST-1 (Basel, Switzerland) colorimetric assay can be used. The incubation lasted for 2 h and the absorbance at 450 nm is measured with reference to 620 nm in a microplate reader [108].

#### 2.3.5. Degradation Analysis

Gels as tissue engineering scaffolds are mostly capable of degradation and ultimately absorbed in the body [9]. Gels can be degraded by certain chemicals or enzymes. For example, reducing agents such as DTT or GSH can degrade hydrogels containing disulfide linkages [20,23,109]. Some hydrogels made from nature polymer can be generally degraded by particular enzymes, such as hyaluronidases for hyaluronic acid [19] lysozyme for chitosan [33] and collagenase for collagen or gelatin [45]. Cells can also have effects on hydrogel degradation since cells can produce enzymes. For example, alginate hydrogel can be partially degraded by fibroblasts encapsulated in it and release calcium ions from its construct as a result [110]. A lot of methods have been developed to conduct degradation analysis. After degradation, the weight of hydrogel main body will be reduced, so the degradation can be directly characterized by the changes of hydrogel weight [93]. Since some degradation components will also be released from the gel main body into the testing solution, another qualitative approach to characterize the gel degradation is showing the release profile of the degradation component by collecting the degradation component and then calculating the cumulative amount of this component [111]. For detecting local degradation in gel systems, some researchers incorporate a FRET(fluorescence resonance energy transfer)-based linker within the gel-forming macromers [8]. Once the gel is treated by degradation solution, the FRET-based linker will generate strong fluorescence where the cells will subsequently extend the process and move within the degraded gel, leaving tracks of increased fluorescence. Upon local gel degradation by encapsulated fibroblasts, the increased fluorescence is observed, allowing visualization of the degradation tracks generated by the migrating cells degradable linker within the PEG-based gel [45]. 

#### 2.3.6. Chemical Properties

Characterization of chemical properties includes clarification of macromer molecular weight that can be measured by Gel permeation chromatography (GPC), analysis of chemical structure by NMR [36], and so on. Among these, gelation efficiency is the important parameter that describes the capability of a hydrogel and can be expressed by the following equation:

*G_f_*= *W_d_*/Wp*100% (7)

where *W_d_* is the weight of the dry hydrogel and Wp is the total feed weight of the two macromer precursors and the photo-initiator [104].

## 3. Bone TE Application

A number of papers have reviewed the application of hydrogels, concerning such aspects as drug delivery, biosensors, catheters, and sutures, contact lenses, bum dressings, and dentures, and artificial organs [7,10,11]. Many of these applications have focused the use of hydrogels as scaffolds to regulate and control cell proliferation and differentiation for the construction of artificial organs. This is especially the case of stem cells or some types of adult cells able to assist proliferation in tissues or organs. A clarification of such cases, and of the various approaches involved, may have to be stated in genetic terms, which are necessary especially for uninitiated students and new researchers. The following will be such a statement. 

There are two main approaches for bone regeneration in tissue engineering: use of autogenetic (*i.e.*, autogenetic bone grafts) and synthetic materials, that is, the cell-free and cell-based approaches (although none of them has translated to clinical practice [112]). Biomaterials for bone regeneration have developed from ceramic, metal materials to such tissue engineering materials as nanofiber and hydrogel [113,114]. Most of them are based on a bone matrix component such as calcium phosphate, hydroxyapatite, silica, and collagen/gelatin, which provide the mechanical and biochemical environment necessary for bone regeneration and can be fabricated with high structural resolution [114]. 

Natural hydrogels such as matrigel, alginate or agarose gel, gelatin/collagen, and synthetic hydrogels such as PEG and PLA/PGA containing cell attachable biotin have often been used for bone tissue engineering research [114,115], but their clinic usage as bone regeneration biomaterials is still limited [112]. Currently, the main problems in bone regeneration tissue engineering are those that relate fate regulation of stem cells, improvement of mechanical properties, vascularization, swelling after injection, and toxicity originating from unreacted constituents [112,116,117,118].

For stem cell fate regulation, it has been reported that the local matrix stiffness on cell state has important implications for development, differentiation, disease, and regeneration [119]. In order to control stem cell fate, researchers have examined the stiffness effect on stem cells by comparing the cell/matrix interaction between 2D and 3D in alginate hydorgel. They demonstrate the commitment of mesenchymal stem-cell population’s changes in response to the rigidity of three-dimensional microenvironments, with osteogenesis (bone differentiation) occurring predominantly at 11–30 kPa. Hydrogels for this study are conjugated with RGD to help cell settlement and attachment. The genes used for characterization of osteogenic differentiation are osteogenic biomarkers core binding factor 1 (Cbfa-1), osteopontin (OPN) and osteocalcin (OCN) [40].

2D surface properties regulating stem cells fate is another aspect commonly investigated, as more and more evidence shows spatial control based on surface morphology could control stem cell differentiation. Some researchers tried controlling stem cell morphology and differentiation by hydrogel surface wrinkles, and found that cells attaching to the lamellar pattern will spread by taking the shape of the pattern, exhibiting high AR, and getting differentiated into an osteogenic phenotype. In contrast, cells attaching inside the hexagonal patterns remain rounded with low spreading before differentiating into an adipogenic phenotype [117]. But it is important to be aware of the highly strict requirement of experimental design to control the single variable in an experiment because there are too many variables which could affect the stem cell differentiation. Effects of other factors, such as materials’ surface wetability and cell adhesion composition, are still unclear [120].

Characterization methods for bone differentiation like Von Kossa-stained histological sections [121], Energy-dispersive X-ray (EDX) analysis of spherulitic minerals and rhombohedral minerals [39], Alizarin Red S staining and Alkaline phosphatase assay [122] are widely used.

The ultimate goal of bone tissue engineering is, very likely, to understand how chemical and physical environments regulate osteogeneis of stem cells, osteoblast, and osteoclasts. Using genetic approaches, more and more genes and their expression pathways may be researched, with increasing interest in the control of cell proliferation and differentiation by referring to hydrogel properties.

## 4. Summary

We have reviewed hydrogels from various preparations (materials and gelation method) and characterization processes, focusing on their physical and chemical properties and how these are related to their function when applied in tissue engineering. However, their successful applications in the related research and development areas far from match the popular interests and demands in biomedicine, gene therapy, and healthcare in general. This review is intended to give introductory information to new researchers who are to work on tissue engineering biomaterials and scaffolds based on hydrogels. 

Research in tissue engineering requires good understanding on scaffolding materials as well as their interactions with cells, tissues, and organs. Some common yet critical issues include encapsulated cell viability in scaffolds after implantation, cell-fate control, and tissue differentiation control. Solutions to these problems will be the result of close collaborations among scientists from multiple disciplines. The future efforts of research on tissue engineering scaffolds based on hydrogels shall aim at having better controllable biomimic materials and more efficient approaches, and being a step closer to their clinical applications. 
